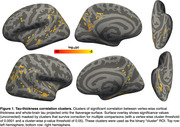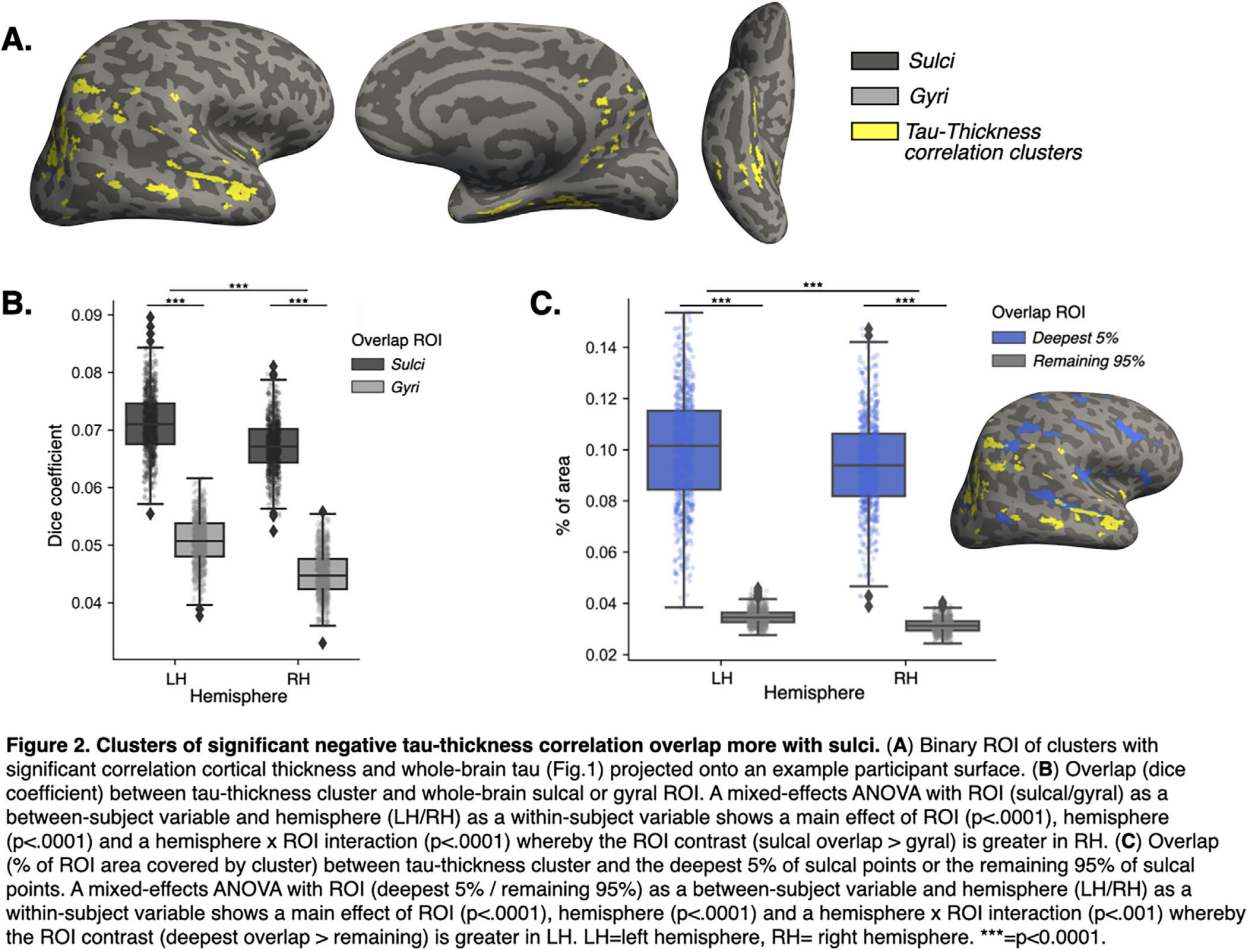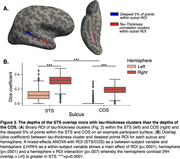# Tau‐related cortical thinning is concentrated in sulcal depths

**DOI:** 10.1002/alz.095011

**Published:** 2025-01-09

**Authors:** Samira A. Maboudian, Benjamin J. Parker, Kevin S. Weiner, William J. Jagust

**Affiliations:** ^1^ University of California, Berkeley, Berkeley, CA USA; ^2^ Lawrence Berkeley National Laboratory, Berkeley, CA USA

## Abstract

**Background:**

Prior studies suggest sulci are more vulnerable to age‐ and Alzheimer’s disease (AD)‐related atrophy than gyri, but the pattern of regional vulnerability to tau pathology is incompletely understood. We performed this study to determine whether tau‐related atrophy preferentially affects sulci and specifically the early‐developing, deepest portions of sulci, which have been shown to be connectivity hubs.

**Methods:**

We used MRI, amyloid and tau PET scans from 809 Alzheimer’s Disease Neuroimaging Initiative participants: 92 with AD, 249 with MCI, 468 cognitively normal. Correlation between vertex‐wise cortical thickness and whole‐brain tau was performed using FreeSurfer’s GLM pipeline. Following cluster‐wise multiple comparisons correction, an ROI of significant clusters was generated (“clusters;” Fig. 1). Overlap between the clusters and whole‐brain sulcal or gyral ROIs was calculated using the dice coefficient. We then generated labels of the deepest 5% of points in the sulcal ROI or the remaining 95%, as well as the deepest 5% of points of the superior temporal sulcus (STS) and collateral sulcus (COS, chosen as a control), and calculated their overlap with the clusters. For all comparisons we used mixed‐effects ANOVAs to examine the effects of ROI (sulcal/gyral, deeper/shallower points, STS/COS) and hemisphere (left/right) on the amount of overlap with the clusters.

**Results:**

Clusters of significant negative tau‐thickness correlation overlap significantly (p<.0001) more with sulci than gyri (Fig. 2B). This overlap is also significantly (p<.0001) greater in the deepest sulcal points compared to the rest of the sulci (Fig. 2C). This effect was greater in the STS than COS (Fig. 3). There is a right hemisphere (RH) bias in this overlap, which is stronger for the STS.

**Conclusions:**

We find that tau‐related thinning occurs mostly in sulci, and particularly in sulcal depths. This overlap is particularly striking within the STS, a brain region particularly vulnerable to AD pathology and deeper in the RH. These results show greater specificity for sulcal vulnerability in aging and AD, particularly in the deepest parts of sulci. This anatomical specificity may underlie the profound effects of tau deposition on cognition.